# Levonorgestrel intrauterine system associated amenorrhea: a systematic review and metaanalysis

**DOI:** 10.1016/j.ajog.2018.12.008

**Published:** 2019-05

**Authors:** Jill E. Sergison, Lauren Y. Maldonado, Xiaoming Gao, David Hubacher

**Affiliations:** a FHI 360, Contraceptive Technology Innovation, Durham, NC; b FHI 360, Biostatistics, Durham, NC; c UNC Gillings School of Global Public Health, Department of Maternal and Child Health, Durham, NC

**Keywords:** amenorrhea, levonorgestrel intrauterine system

## Abstract

**Objective Data:**

Amenorrhea is a polarizing noncontraceptive effect of the levonorgestrel intrauterine system. Composite amenorrhea prevalence estimates that summarize all clinical data for the first-year after insertion currently are not available. The purpose of this study was to investigate the validity of existing prevalence estimates by the systematic calculation of amenorrhea measures for a general population of levonorgestrel intrauterine system users and to provide 90-day interval point estimates for the first year of use.

**Study:**

We identified clinical trials, randomized controlled trials, and randomized comparative trials that were published in English between January 1970 and September 2017 through electronic searches of 12 biomedical and scientific literature databases that included MEDLINE and ClinicalTrials.gov.

**Study Appraisal and Synthesis Methods:**

We considered studies that clearly defined amenorrhea per World Health Organization standards (the complete cessation of bleeding for at least 90 days), collected data from written daily bleeding diaries (the gold standard data collection technique on menstrual bleeding changes), and evaluated levonorgestrel intrauterine system devices that released 20 μg of levonorgestrel per day. We assessed study quality using guidelines established by the US Preventive Services Task Force and Cochrane handbook for systematic reviews of interventions. Two reviewers independently conducted all review stages; disagreements were resolved by a third reviewer. Where possible, data were pooled with the use of a random-effects model.

**Results:**

Of 2938 potentially relevant studies, we included 9 in our meta-analysis. We calculated amenorrhea prevalence, which was weighted for inter- and intrastudy variance, for 4 90-day intervals and months 0–12. Our results demonstrated few levonorgestrel intrauterine system users (0.2%; 95% confidence interval, 0.0–0.4) experienced amenorrhea during the first 90 days after insertion; however, prevalence increased to 8.1% (95% confidence interval, 6.6–9.7) on days 91–180. Finally, 18.2% (95% confidence interval, 14.9–21.5) of users experienced amenorrhea for at least 1 90-day interval during the first year. Although interstudy heterogeneity limited reliability of days 181–271 and 272–365 measures, prevalence increased from 13.6% (95% confidence interval, 9.3–18.0) to 20.3% (95% confidence interval, 13.5–27.0), respectively.

**Conclusion:**

Approximately 20% of levonorgestrel intrauterine system users experience amenorrhea during at least 1 90-day interval by the first year after insertion. This composite estimate is consistent with the product labeling and demonstrates that most users do not experience amenorrhea during the first year. These results provide accurate summary measures to facilitate counselling and informed method selection.

When counseling potential and existing levonorgestrel intrauterine system (LNG-IUS) users, providers underscore possible menstrual bleeding pattern changes` because the changes are often associated with hormonal contraceptive use. Amenorrhea, defined by the World Health Organization (WHO) as the complete absence of bleeding or spotting for 90 consecutive days, is 1 of the LNG-IUS’s potential, albeit polarizing, noncontraceptive features.^1^, ^2^ Although amenorrhea is consistently among the most commonly cited reasons for LNG-IUS discontinuation, recent studies suggest some women intentionally may seek methods that are associated with reduced bleeding because of potential noncontraceptive health and lifestyle advantages.^3^, ^4^, ^5^, ^6^ As such, accurate counseling on amenorrhea is crucial to adequately address diverse patient interests and concerns related to bleeding changes with this method.

**AJOG at a Glance**

**Why was this study conducted?**

The purpose of this study was to investigate the validity of existing amenorrhea prevalence estimates by systematically calculating first-year measures for a general population of levonorgestrel intrauterine system users and provide new 90-day interval estimates to guide accurate counselling and informed method selection.

**Key findings**

Our findings are consistent with estimates in the existing levonorgestrel intrauterine system product labels and demonstrate that most users do not experience amenorrhea during the first year of use.

**What does this add to what is known?**

Our study provides composite amenorrhea prevalence estimates that were derived from all available clinical data during the first-year after insertion. We additionally provide 90-day interval measures to facilitate counseling on the timeframe over which this occurs. Regardless of whether potential users desire or dislike amenorrhea, accurately establishing expectations with the levonorgestrel intrauterine system may improve informed method selection that aligns with individual needs.

In the 1980s, the WHO established strict definitions and data collection techniques, which included the implementation of written daily menstrual diaries, for characterizing contraceptive-associated bleeding changes.^2^ Clinical trials for both Mirena (Bayer, Whippany, NJ) and Liletta (Allergan Inc., Irvine, CA), the 2 most prevalently used LNG-IUS devices, adhered to these guidelines. Their product labels, which cite that 18.4% of Mirena users and 19.0% of Lilleta users experience amenorrhea by the end of the first year of use, currently serve as the basis for counselling on menstrual changes that are associated with these methods.^7^, ^8^

Since the introduction of these 2 products in the early 2000s, however, several other clinical trials on the LNG-IUS have been published. Additionally, data from past trials on LNG-IUS devices with the same hormonal concentration and release profile are not captured in estimates provided in the Mirena and Liletta labelling. Amenorrhea data from these trials may impact prevalence estimates, and a single source that summarizes all available clinical data on this outcome during first-year use currently does not exist. Further, additional information on amenorrhea prevalence per 90-day interval comprising the first year of use may improve counselling on the timeframe over which this menstrual change occurs.

## Objective

We systematically reviewed the literature and calculated composite amenorrhea prevalence measures for a general population of LNG-IUS users throughout the first year after insertion. In doing so, we aimed to investigate the validity of existing prevalence estimates in the Mirena and Liletta product labeling and to add 90-day interval point estimates during the first year of use.

## Methods

### Eligibility criteria, information sources, search strategy

**Figure A1:**
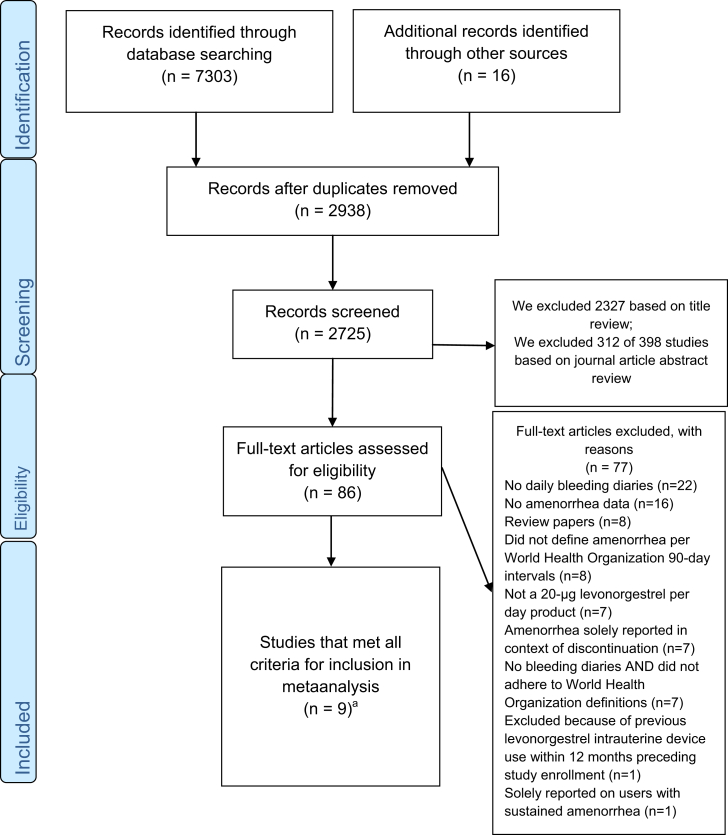
Study selection process The selection process followed the Preferred Reporting Items for Systematic Reviews and Meta-analysis guidelines. ^a^Studies included in 90-day interval analysis (n=6); studies included in 0–12 month analysis (n=3). *Sergison and Maldonado. Amenorrhea associated with LNG-IUS use. Am J Obstet Gynecol 2019*.

We conducted this systematic review in accordance with the reporting guidelines outlined in the Preferred Reporting Items for Systematic Reviews and Meta-Analyses (PRISMA) Statement.^9^ Studies published in English, between January 1970 and September 2017, were eligible for review. We searched for relevant published literature using various electronic biomedical and social science literature databases, including MEDLINE, PubMed, SCOPUS, Popline, CINAHL, PsycInfo, Web of Science, Global Health, Academic Search Premier, Africa Wide Info, African Index Medicus, and registered trials on ClinicalTrials.gov. A librarian who had experience in systematic review searching developed our PubMed search strategy, and another librarian peer-reviewed the strategy using the peer review of electronic search strategy standard. We applied the full PubMed strategy to all databases with modifications to search terms as necessary (Figure A1). We additionally searched Google and Google Scholar for relevant gray literature (ie, literature not formally published in sources such as books or journal articles) not captured in our database search.^10^ We conducted our last search on September 15, 2017.

Our outcome of interest was the prevalence of amenorrhea that was associated with LNG-IUS use throughout the first-year after insertion. Search strategies combined keywords such as “amenorrhea,” “bleeding pattern,” and “menstrual changes” that were specific to LNG-IUS use. Of note, we did not exclude copper intrauterine devices or other contraceptive methods from our search terms so as not to eliminate comparative trials with LNG-IUS data.

### Study selection

We considered clinical studies, which included clinical trials, randomized controlled trials, and randomized comparative trials, if they met the following criteria: reported data on LNG-IUS devices release 20 μg of levonorgestrel (LNG) per day; defined amenorrhea explicitly as 90 consecutive days without bleeding or spotting; and collected menstrual bleeding data from written daily diaries. We chose to evaluate the 20-μg levonorgestrel release product because it has been studied extensively and is currently the most widely used hormonal intrauterine device in the United States. We included women of any parity, age, race, or ethnicity. We excluded studies on women with recent pregnancy (ie, within 6 months postpartum, postabortion, or breastfeeding) or with a history of LNG-IUS use within 12 months preceding enrollment, because women with these conditions are more likely to experience menstrual bleeding pattern changes.^11^, ^12^, ^13^, ^14^ We excluded studies that reported amenorrhea data solely in the context of reasons for method discontinuation. Finally, we excluded papers on women with heavy menstrual bleeding because of structural (ie, uterine fibroid tumors) or hormonal disease because the LNG-IUS differentially impacts menstruation depending on baseline blood loss volume.^15^ Further, numerous systematic reviews on treating heavy menstrual blood loss with the LNG-IUS have been published already.^16^, ^17^, ^18^

Two independent reviewers (J.E.S., L.Y.M.) performed the study selection process using specific inclusion criteria to ensure accuracy and reproducibility. The first screening included all titles and abstracts of identified publications; we retrieved all potentially relevant studies for full-text evaluation. Both reviewers independently evaluated full-text articles and recorded reasons for exclusion. At each step in the review process, a third reviewer (D.H.) resolved any disagreements. If we identified duplicate studies, we selected either the most recent or most complete publication. Our study selection process is presented in the Figure A1.

### Data extraction

Two investigators (J.E.S., L.Y.M.) independently extracted amenorrhea data from articles that were selected for inclusion using standardized data extraction sheets. We recorded data on study characteristics such as study design, location, population, exposure and outcome measurements, participant characteristics, duration of follow up, and adjustment in analyses. We extracted amenorrhea prevalence data that were collected from bleeding diaries during the first-year after insertion for each 90-day interval or reported as a single 0–12 month estimate. We defined amenorrhea prevalence as the proportion of women who reported amenorrhea (complete absence of bleeding or spotting for at least 90 days) among total completed bleeding diaries that were assessed for each interval. Studies typically reported amenorrhea data in either tabular or graphic form. In instances in which graphic data were not depicted with a numeric point estimate, both investigators directly measured the estimate from the graph using a computer-generated right-angle ruler. In discrepant cases, the investigators calculated and included the mean of the 2 extracted values in the final recorded estimate.

### Critical appraisal process

We critically appraised studies based on guidelines established in the US Preventive Services Task Force procedure manual and the Cochrane handbook for systematic reviews of interventions.^19^, ^20^ These resources guide investigators through the process of appraising studies through careful evaluation of both study bias and quality. We assessed study bias based on the likelihood of attrition. We deemed study bias “low” if attrition was <10%. Similarly, we deemed study bias “medium” and “high” if attrition was at 10–20% or >20%, respectively. We assessed study quality by study location (multiple vs single country), data presentation (tabular vs graphic depiction of point estimate), and the consistency of interval data provided (provided data for all 4 90-day intervals or only certain intervals). We assumed tabular data were more accurate, thus of higher quality, than graphic data (without accompanying numeric point estimates) because of potential for human error in manually estimating measures. We accounted for sample size in our meta-analysis; thus, size was not considered in the critical appraisal process. We did not exclude studies based on quality or bias alone. Details of our critical appraisal are presented in Appendix B.

### Data synthesis

We pooled amenorrhea prevalence estimates for the following intervals: days 0–90, 91–180, 181–271, 272–365 and months 0–12. We used a random-effects model to account for variance in data pooled to create our composite measures. We generated this model by incorporating estimates weighted for inter- and intrastudy variance.^21^ We used 2 measures to determine the degree of heterogeneity in our meta-analysis: the Q-statistic for which a probability value of <.10 was interpreted as statistical evidence of heterogeneity and the I^2^ statistic (range, 0–100%) and its 95% confidence interval (95% CI). We considered I^2^ values <50% to be evidence of mild-to-moderate heterogeneity.^22^ Interstudy heterogeneity reflects the variance in results contributed by included studies, which may be attributable to differences in study population, design, analysis technique, among other characteristics. If results for any given interval exhibited significant interstudy heterogeneity, we conducted additional sensitivity analyses by excluding potential outliers and presented new prevalence estimates, I^2^ values, and Q-statistic probability values. We weighted prevalence estimates for inter- and intrastudy variance by calculating precision-based (inverse variance) weights.^21^ Specifically, the weight for each study was determined by the reciprocal of the sum of the intra- and interstudy variance per 90-day interval (formula in Appendix A2). As such, studies with less variance or greater precision were ascribed greater weight in the calculation of prevalence estimates. We applied this weighting scheme to standardize our interval estimates and thereby increase generalizability of our results. We conducted all analyses in SAS software (version 9.4; SAS Institute, Cary, NC). Our review and meta-analysis did not involve experimentation on human subjects and thus did not necessitate institutional review board review.

## Results

### Study selection

Our initial literature search resulted in 2938 unique titles, of which we deemed 86 full-text articles relevant to our research question after title and abstract review. Nine articles met full criteria for inclusion in our metaanalysis (Figure A1). We excluded 22 studies for lack of daily bleeding diary data, 16 studies for lack of amenorrhea data, 8 review papers with redundant data, 8 studies that did not adhere to WHO definitions, 7 studies that evaluated products that did not release 20 μg of levonorgestrel per day, 7 studies for reporting solely on amenorrhea in the context of method discontinuation, 7 studies that did not use bleeding diaries nor adhere to WHO-definitions, and 1 study that evaluated participants who underwent LNG-IUS reinsertion in <12 months before discontinuing their previous device. Last, we excluded 1 study that reported prevalence data for users with sustained amenorrhea, because this composite measure excluded users who may have resumed bleeding or spotting after a 90-day period without bleeding.^23^

### Study characteristics

Specific details that include the design, population, prevalence point estimates, and strengths and weaknesses of each study are summarized in Appendix B. Among the 9 studies included in our analysis, 7 studies were randomized comparative trials and 2 studies were noncomparative cohort studies (clinical trials). Study participants were recruited from 14 countries spanning 5 continents, namely: Finland, Sweden, Denmark, Hungary, Norway, the United States, the United Kingdom, France, China, Brazil, Egypt, Chile, Singapore, and the Dominican Republic. Further, study participants ranged from ages from 16–45 years; 1 study evaluated a slightly older group of LNG-IUS users, ages 35–45 years.^24^ Two studies presented measures from the same study cohort, 1 presenting data in 90-day intervals and the other as a single 0–12 month prevalence.^25^, ^26^ Four studies reported prevalence data for all 4 90-day intervals.^26^, ^27^, ^28^, ^29^ Two studies solely reported data for the second interval (days 91–180).^30^, ^31^ Three studies presented a single 0–12 month estimate of the proportion of users who experienced amenorrhea for any 90-day interval during the first year after insertion.^24^, ^25^, ^32^ Last, 2 studies presented 0–6 month composite prevalence measures, but we chose not to pool these data in our analysis because of significant interstudy heterogeneity.^24^, ^25^

### Risk of bias of included studies

Our quality assessment is detailed in Appendix B. Four studies received a score of “good” quality, and 5 studies received a score of “fair.” We reported “low” bias for 3 studies, “medium” bias for 2 studies, and “high” likelihood of bias for 4 included studies. Because study bias was largely driven by attrition, we chose not to eliminate any study on the likelihood of bias alone.

### Synthesis of results

**Figure 1:**
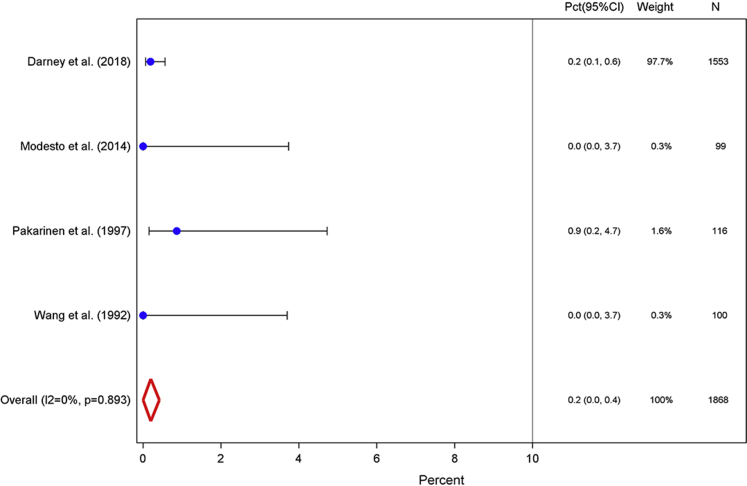
Days 0–90 Weighted percentage of levonorgestrel intrauterine system users who experienced amenorrhea, defined as the proportion of users who reported complete cessation of bleeding or spotting for at least 90 days among total users with completed menstrual bleeding diaries, for the first interval after insertion. *CI*, confidence interval; *Pct*, percentage. *Sergison and Maldonado. Amenorrhea associated with LNG-IUS use. Am J Obstet Gynecol 2019*.

Our weighted amenorrhea prevalence measures across all 5 analyzed intervals are summarized in the Table. Four studies contributed bleeding diary data from 1868 study participants for the first 90-day interval (days 0–90).^26^, ^27^, ^28^, ^29^ The weighted proportion of users who experienced amenorrhea in the first 90 days after insertion was 0.2% (95% CI, 0.0–0.4; Figure 1). These 4 contributing studies presented very similar point estimates, and only 4 LNG-IUS users experienced complete absence of bleeding or spotting during this period. This pooled measure yielded an I^2^ value of 0.0% and a corresponding probability value of .893 and indicated homogeneity across studies.

**Table:** Summary of weighted amenorrhea prevalence measures in percentage of levonorgestrel intrauterine system users for 4 90-day intervals and the first year after insertion

Interval	Total participants across studies, n	Random effect, % (95% confidence interval)	I^2^, % (95% confidence interval)	Included studies, n	Degrees of freedom	*P* value
Days						
0–90	1868	0.2 (0.0–0.4)	0.0	4	3	.893
91–180	2748	8.1 (6.6–9.7)	37.6 (0.0–75.2)	6	5	.155
181–271	1665	13.6 (9.3–18.0)	64.0 (0.0–87.8)	4	3	.039
272–365	1565	20.3 (13.5–27.0)	76.4 (35.4–91.4)	4	3	.005
Month 0–12	1740	18.2 (14.9–21.5)	30.6 (0.0–76.2)	3	2	.237

*Sergison and Maldonado. Amenorrhea associated with LNG-IUS use. Am J Obstet Gynecol 2019*.

**Figure 2:**
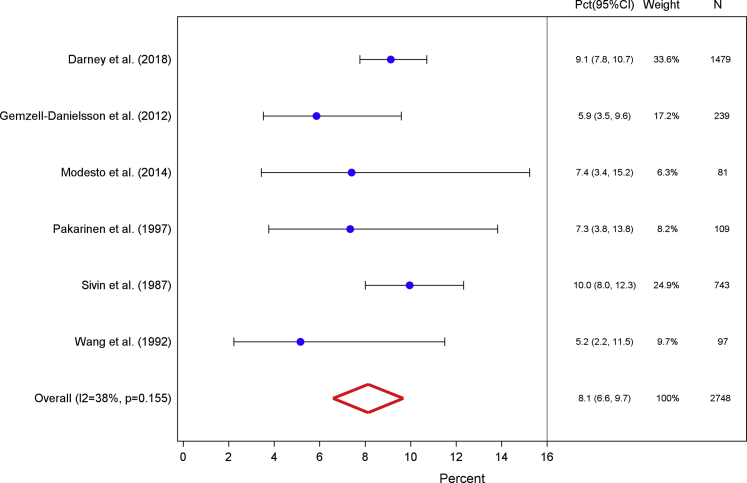
Days 91–180 Weighted percentage of levonorgestrel intrauterine system users who experienced amenorrhea, defined as the proportion of users who reported complete cessation of bleeding or spotting for at least 90 days among total users with completed menstrual bleeding diaries, for the second interval after insertion. *CI*, confidence interval; *Pct*, percentage. *Sergison and Maldonado. Amenorrhea associated with LNG-IUS use. Am J Obstet Gynecol 2019*.

We pooled data from 2748 participants across 6 studies to calculate the second amenorrhea prevalence for days 91–180 after insertion.^26^, ^27^, ^28^, ^29^, ^30^, ^31^ The weighted proportion of LNG-IUS users who experienced amenorrhea during the second 90-day interval increased to 8.1% (95% CI, 6.6–9.7; Figure 2). Studies pooled in this measure were mildly heterogeneous, with an I^2^ value of 37.6% and Q-statistic probability value of .155. Two studies contributed more than one-half the bleeding diary data for this calculation and reported the greatest proportion of users with amenorrhea in the second 90-day interval.^27^, ^31^ Additionally, when we restricted our analysis solely to the 4 studies that contributed data for all 4 90-day intervals, our results did not significantly change (analyses not shown).

**Figure 3:**
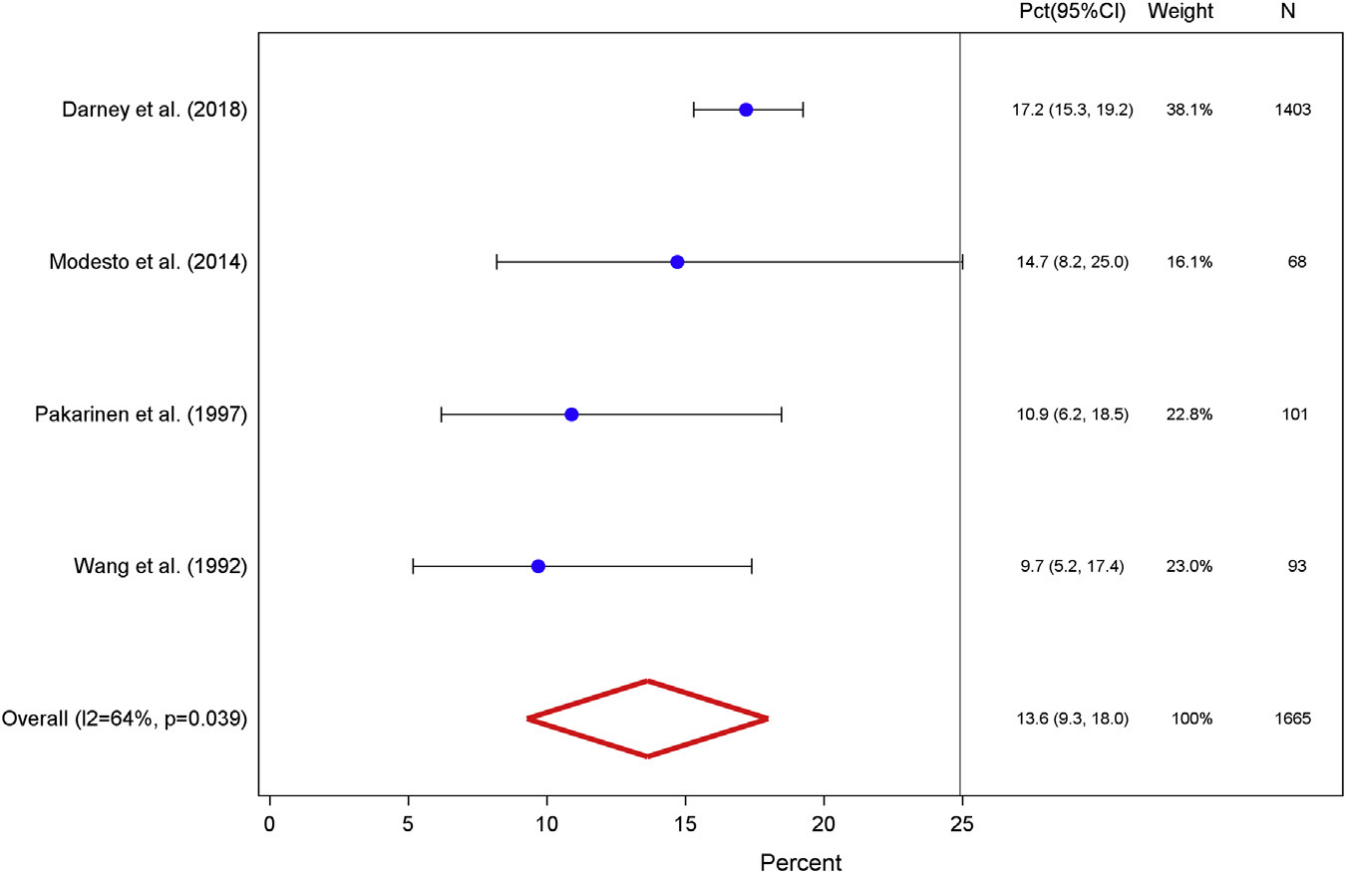
Days 181–271 Weighted percentage of levonorgestrel intrauterine system users who experienced amenorrhea, defined as the proportion of users who reported complete cessation of bleeding or spotting for at least 90 days among total users with completed menstrual bleeding diaries, for the third interval after insertion. *CI*, confidence interval; *Pct*, percentage. *Sergison and Maldonado. Amenorrhea associated with LNG-IUS use. Am J Obstet Gynecol 2019*.

**Figure A2:**
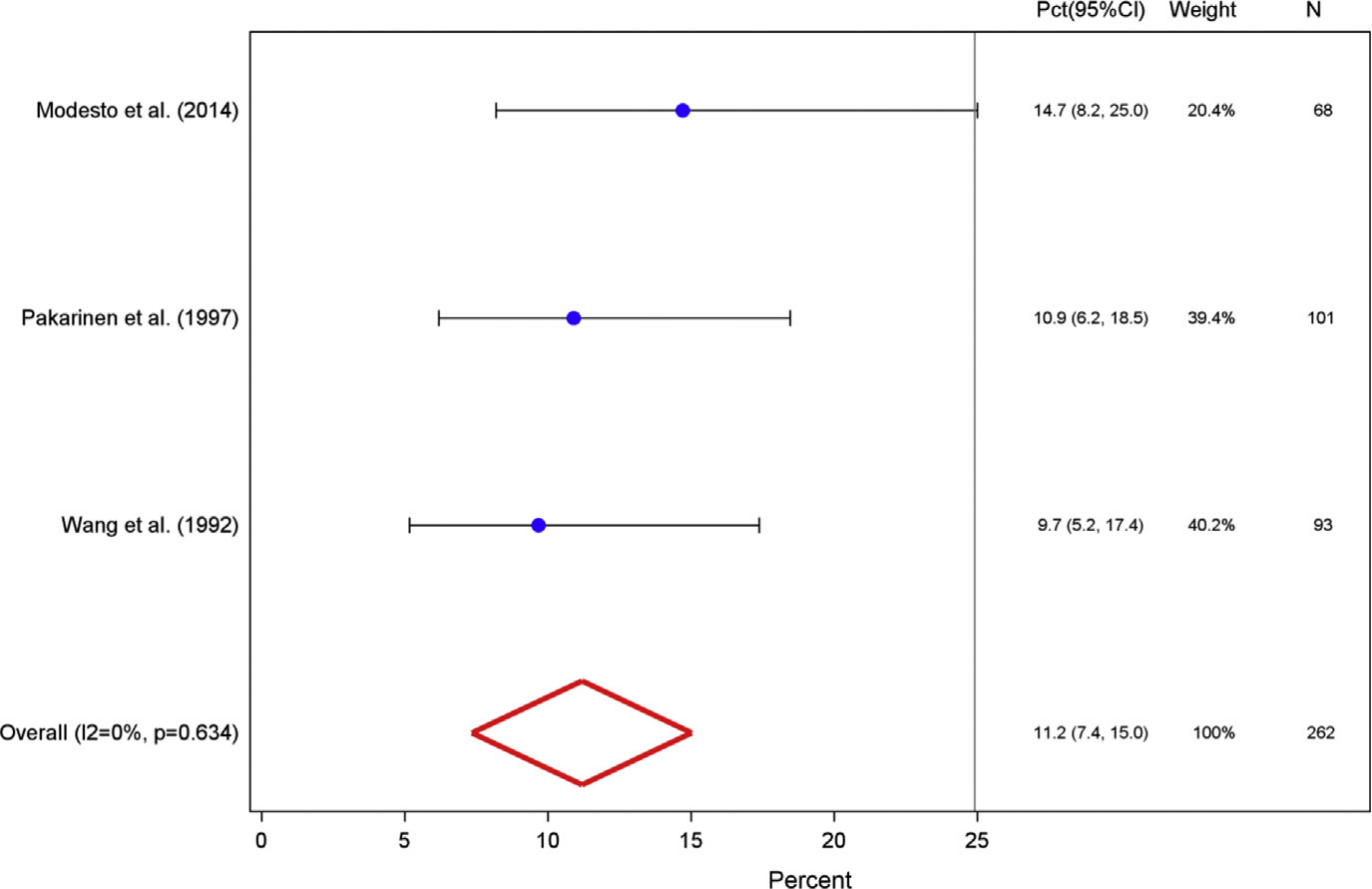
Days 181–271 Weighted percentage of levonorgestrel intrauterine system users who experienced amenorrhea, excluding Darney et al,^27^ for the third interval after insertion. *CI*, confidence interval; *Pct*, percentage. *Sergison and Maldonado. Amenorrhea associated with LNG-IUS use. Am J Obstet Gynecol 2019*.

Four studies contributed data from 1665 diaries to calculate the weighted proportion of users who experienced amenorrhea during days 181–271 after insertion.^26^, ^27^, ^28^, ^29^ The prevalence of amenorrhea during the third 90-day interval was 13.6% (95% CI, 9.3–18.0; Figure 3). The studies that were included in this calculation exhibited significant heterogeneity with an I^2^ value of 64% and Q-statistic probability value of .039. A follow-up sensitivity analysis that removed outlying data yielded a prevalence of 11.2% (95% CI, 7.4–15.0), with a corresponding I^2^ value of 0% and Q-statistic probability value of .634 (Figure A2).^27^

**Figure 4:**
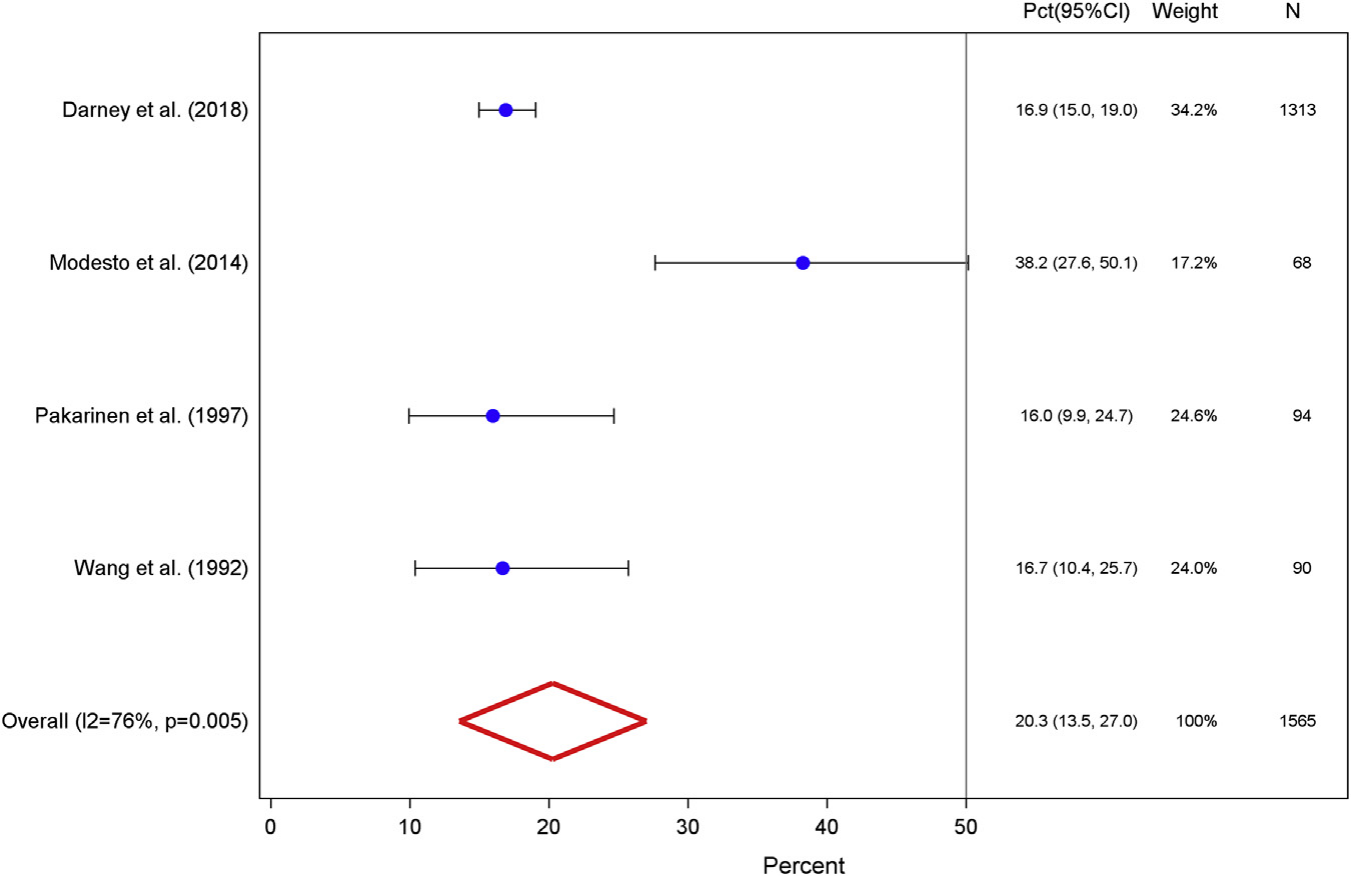
Days 272–365 Weighted percentage of levonorgestrel intrauterine system users who experienced amenorrhea, defined as the proportion of users who reported complete cessation of bleeding or spotting for at least 90 days among total users with completed menstrual bleeding diaries, for the fourth interval after insertion. *CI*, confidence interval; *Pct*, percentage. *Sergison and Maldonado. Amenorrhea associated with LNG-IUS use. Am J Obstet Gynecol 2019*.

**Figure A3:**
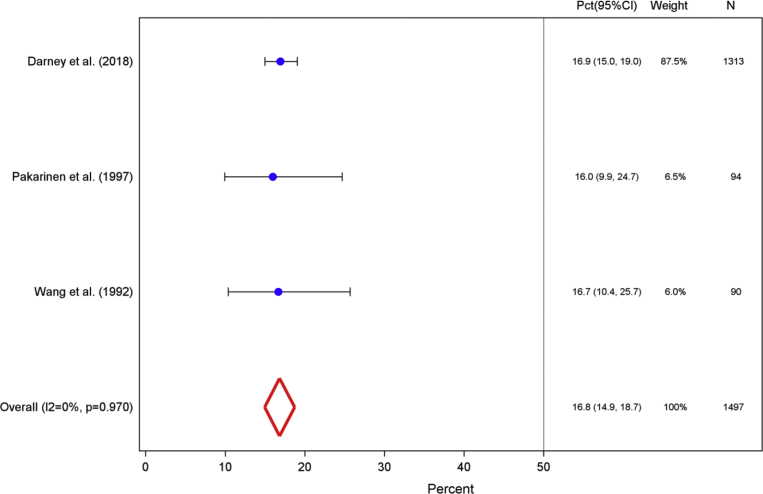
Days 272–365 Weighted percentage of levonorgestrel intrauterine system users who experienced amenorrhea, excluding Modesto et al,^28^ for the fourth interval after insertion. *CI* confidence interval; *Pct*, percentage. *Sergison and Maldonado. Amenorrhea associated with LNG-IUS use. Am J Obstet Gynecol 2019*.

Bleeding diary data from 1565 participants, pooled across 4 studies, yielded a final 90-day interval (days 272–365) prevalence measure of 20.3% (95% CI, 13.5–27.0).^26^, ^27^, ^28^, ^29^ The I^2^ value of 76.4% and Q-statistic probability value of .005 that correspond to this measure reveal marked heterogeneity across studies (Figure 4). Of note, Modesto et al^28^ reported a notably high amenorrhea prevalence of 38.2% (95% CI, 27.6–50.1). When we excluded these data in a subsequent sensitivity analysis, we calculated a lower weighted amenorrhea prevalence of 16.8% (95% CI, 14.9–18.7) with a corresponding I^2^ value of 0% and Q-statistic probability value of .970 (Figure A3).

**Figure 5:**
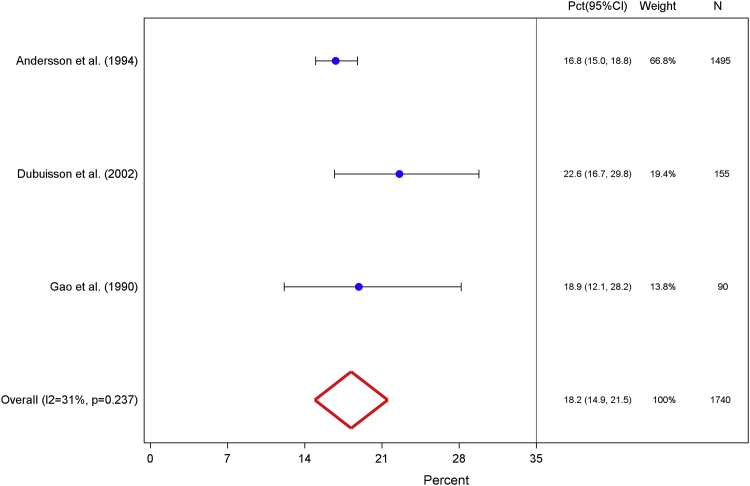
Days 0–365 Weighted percentage of levonorgestrel intrauterine system users who experienced amenorrhea, defined as the proportion of users who reported complete cessation of bleeding or spotting for at least 90 days among total users with completed menstrual bleeding diaries, for any 90-day interval during the first 12 months after insertion. *CI*, confidence interval; *Pct*, percentage. *Sergison and Maldonado. Amenorrhea associated with LNG-IUS use. Am J Obstet Gynecol 2019*.

Finally, 3 studies reported 0–12 month amenorrhea prevalence data from bleeding diaries of 1740 participants.^24^, ^25^, ^32^ Our calculated amenorrhea prevalence during any 90-day interval throughout the first year after LNG-IUS insertion was 18.2% (95% CI, 14.9–21.5; Figure 5). The corresponding I^2^ value of 30.6% and Q-statistic probability value of .237 revealed mild interstudy heterogeneity.

## Comment

### Main findings

In this systematic review and metaanalysis, we summarized available data from clinical trials, randomized controlled trials, and randomized comparative trials and derived pooled measures of amenorrhea prevalence for a general population of reproductive-aged LNG-IUS users. Our objective was to provide clinicians and clients with reliable prevalence measures to guide accurate counselling and method selection.

We observed a paucity of high-quality data that quantified experiences with amenorrhea for the LNG-IUS. Although our literature search produced thousands of results, only 9 studies ultimately met inclusion criteria for our metaanalysis. We excluded some studies because they failed to define amenorrhea explicitly per WHO standards.^15^, ^33^ For example, a few studies reported cumulative prevalence measures for the first 6 months, rather than in discrete intervals.^15^, ^33^ Another study reported amenorrhea prevalence at single points in time but did not state the interval covered by each estimate or whether information was recorded prospectively by participants.^34^

Amenorrhea prevalence measures for the latter 90-day intervals comprising months 6–12 after insertion revealed significant interstudy heterogeneity among the 9 studies included in this systematic review. When we excluded studies that contributed outlying amenorrhea prevalence data in our sensitivity analyses, our results yielded prevalence measures of 11.2% and 16.8% with mild heterogeneity for the third (days 181–271) and fourth (days 272–365) intervals, respectively. When results for these latter 2 intervals are considered in conjunction with our measures for the first 2 90-day intervals, we observe an upward trend in the proportion of users who experienced amenorrhea, beginning with a mere 0.2% of users for the first 90 days, followed by 8.1%, 11.2%, and 16.8% in the latter 3 90-day intervals, respectively. The most significant change in the proportion of users who reported amenorrhea occurred between the first and second 90-day interval, with an increase of 7.9%. Finally, our results revealed 18.2% of users reported at least 1 90-day interval of amenorrhea during any period throughout the first year of use. This result is consistent with amenorrhea prevalence measures that are cited in the product labeling for Mirena and Liletta devices of 18.4% and 19.0%, respectively.^7^, ^8^

We attribute the observed interstudy heterogeneity to several possible explanations. First, an LNG-IUS user may be less inclined to note every incidence of spotting, especially if she has not received explicit instructions or reminders to do so from study staff. Further, the inherent challenges of daily diary completion may contribute to heterogeneity in amenorrhea prevalence estimates. The burden of maintaining diaries is not trivial, and participants may experience fatigue or increasing disinterest in diary completion. Previous studies affirm this trend because diary completion tends to decrease over time, which compromises data quality for outcomes reliant on daily, prospective records.^35^ To overcome potential recall bias inherent to written diaries, some studies are transitioning to electronic diaries with lock-out and time-stamp potential to ensure timely, prospective data collection.^36^ Last, because this analysis incorporated data from 14 different countries, we may partially attribute interstudy heterogeneity to differences in bleeding perception and diary recording across cultural contexts. Previous studies demonstrate that there is little consistency in the way women across cultures recall menstrual bleeding changes. This variation is amplified when women are tasked with reporting amenorrhea data because women are more likely to describe occurrences of bleeding days (ie, heavier bleeding, spotting) rather than nonbleeding episodes.^37^ Unfortunately, multicenter studies that were included in our review did not present results by individual country, which limited our ability to stratify results geographically. Future studies may consider addressing this limitation.

### Strengths and limitations

Our systematic review has several strengths. Although bleeding changes that are associated with hormonal contraceptive methods are common, few studies attempt to quantify the prevalence of these outcomes. We used a rigorous approach according to PRISMA guidelines and did not limit our search based on geographic location.^9^ We only included articles if they met our strict criteria, which included the use of daily menstrual diaries (the gold standard for reporting on bleeding outcomes associated with contraceptive methods). Further, we pooled results among studies with mild or moderate heterogeneity for the first 2 90-day intervals. For the latter 2 90-day intervals with high interstudy heterogeneity, we conducted additional sensitivity analyses to demonstrate the effect of removing outlier amenorrhea data. Finally, our review provides composite prevalence estimates that summarize all clinical data on LNG-IUS 20 μg per day products from both before and after the introduction of Mirena and Liletta.

Despite these strengths, our review has several limitations. An intractable limitation that plagues all research in this area is that study subjects’ attrition from trials may bias estimates toward method-related features that are considered favorable by participants.^38^, ^39^ Because intolerable changes in menstrual pattern often lead to product removal, only favorable or tolerable patterns may bias results. Consider the following illustrative example. In the study by Darney et al,^27^ approximately 20% of participants did not complete the first year with the LNG-IUS in situ, as reported in the study’s main paper Eisenberg et al.^40^ If all 20% discontinued because they disliked amenorrhea yet reported a different reason for removal, then conceivably, had they kept the product, the hypothetical amenorrhea prevalence in the last interval could have been as high as 31% (484 of 1575 users), instead of 17% (Appendix B). Further, none of our included studies examined amenorrhea as a primary study outcome. As such, the data collection and reporting of amenorrhea prevalence are often secondary measures. Additionally, it is unlikely that most clinicians or patients consistently characterize amenorrhea according to strict WHO definitions. As a result, anecdotal reports of amenorrhea (broadly defined) may be higher than this conservative analysis demonstrates. Regardless, these conservative point estimates provide the most generalizable expectations of amenorrhea, with the LNG-IUS based on existing clinical data. Finally, the aggregate nature of our data limited our ability to determine whether women consistently experienced amenorrhea across consecutive 90-day intervals. Of note, the article by Nilsson et al^23^ noted that a cumulative 11% of LNG-IUS users experienced amenorrhea during the first 3 months of use and remained amenorrhoeic through the end of 12 months of use. This estimate is nearly 40% lower than our calculated prevalence of 18.2% of users who experienced amenorrhea during any 90-day interval throughout the first year. This discrepancy suggests that many, but not all, women who experience amenorrhea during the first 90-day interval remain amenorrhoeic through subsequent intervals. Future studies should attempt to quantify consecutive amenorrhea rates because this information may benefit both patients and providers.

### Comparison with existing literature

Results of our metaanalysis are consistent with those originally presented in Darney et al,^27^ the prospective multicenter, US-based, phase III clinical trial that contributed data for the Liletta package insert. Bleeding data from this clinical trial were analyzed secondarily and published by Schreiber et al.^41^ Although our findings are consistent with those of previous studies, the added value of deriving amenorrhea estimates through the process of conducting a systematic review and metaanalysis is largely linked to generalizability. Unlike previous studies that examine a US-based population of LNG-IUS users, we broadened our criteria to include studies that were conducted in multiple countries and on all 52-μg LNG-IUS products, which widens the applicability to users internationally and across manufacturers.^42^ Last, the review and analysis of amenorrhea data from multiple studies underscores the importance of comprehensively synthesizing evidence, rather than drawing conclusions from single studies.^43^

### Conclusions and implications

Our review presents reliable amenorrhea prevalence measures for the 4 90-day intervals that comprise the first year of LNG-IUS use. Despite few studies meeting inclusion criteria, our findings summarize existing daily bleeding diary data on this outcome for a general population of all 20 μg per day LNG-IUS users. Further, our findings underscore strengths and limitations of existing studies on amenorrhea prevalence that are associated with the LNG-IUS and other hormonal contraceptives. Future studies are needed that adhere to standardized definitions of menstrual bleeding–related outcomes, examine amenorrhea and other menstrual bleeding changes as a primary outcome of interest, and use rigorous data collection techniques to assure these events are recorded prospectively and frequently.

Overall, our findings are consistent with the existing amenorrhea prevalence estimates that are stated on the Mirena and Liletta product labeling. These results affirm most users will not experience amenorrhea within the first year of LNG-IUS use; however, for the nearly 20% of women that experience this outcome, our 90-day interval measures provide an estimated timeframe over which women may expect this change. Menstrual bleeding changes are a polarizing side-effect of the LNG-IUS and may encourage some women to choose this method and deter others from selection. Regardless, inaccurate expectations of bleeding changes may result in patient concerns and a higher volume of return visits.^44^, ^45^, ^46^ Accurately establishing expectations with the LNG-IUS may improve informed method selection that aligns with individual needs and ultimately decrease discontinuation.

## Figures and Tables

**Appendix B:** Critical appraisal and additional study details for those included in amenorrhea metaanalysis (n=9)

Study (year)	Study design	Study population	Prevalence of amenorrhea per 90-day interval (% of users reporting amenorrhea among total completed bleeding diaries). % (n/N)				Prevalence of amenorrhea for any 90-day interval within first year following insertion (% of users reporting amenorrhea among total completed bleeding diaries), % (n/N)	Strengths	Weaknesses	Quality/bias^a^^,^^b^^,^^c^
			Days 0–90	Days 91–180	Days 181–271	Days 272–365	Days 0–365			
Andersson et al (1994)	Open randomized comparative trial conducted in Finland, Sweden, Denmark, Hungary and Norway; 1987–1992; compared the contraceptive safety and efficacy of LNG-IUS and Nova T copper IUD over 5-years	1821 LNG-IUS users and 937 Nova T copper IUD (Bayer, Whippany, NJ) users; ages 18–38 years; requesting long-acting reversible contraception and not using hormonal contraceptives for 12 months before enrollment were eligible	—	—	—	—	16.8, (251/1495)	Prospective, multinational trial, used validated tool to collect bleeding data, few exclusion criteria, large sample size, long follow-up period, attrition rate <20%	Did not provide estimates for each 90-day interval; only reported prevalence of any 90-day interval without bleeding/spotting in the first year	Good/medium
Darney et al (2018)	Cohort study conducted in the United States, secondary analysis of phase 3 clinical trial; 2013–2014	1566 first-time LNG-IUS users; ages 16–45 years; followed for 12 months after insertion; excluded women who had used a hormonal injectable contraceptive within 9 months of enrollment (or 6 months for those who experienced 2 spontaneous, regular menstrual cycles)	0.2 (3/1553)	9.1 (135/1479)	17.2 (241/1403)	16.9 (222/1313)	—	Prospective trial, used validated tool to collect bleeding data, few exclusion criteria, large sample size, frequent follow-up intervals, attrition rate <20%, separate analysis and reporting for first-time users	Single country study	Good/medium
Dubuisson et al (2002)^d^	Open noncomparative cohort study; France; 2001–2002	203 LNG-IUS users; ages 35–45 years; followed for 12 months afterinsertion; women with at least 1 previous pregnancy, without known LNG-IUS contraindications, and who discontinued use of either an oral contraceptive or Cu-IUD 1–6 months prior due to poor compliance, poor tolerance, or change in risk/benefit ratio were eligibile; assessed completed bleeding diaries at 3, 6, 9, and 12 months, but presented aggregate data for 0–6 and 0–12-month intervals	13.5 (21/155)^d^		—	—	22.6 (35/155)	Prospective study used validated tool to collect daily bleeding data	Limited generalizability because of restricted age range of participants, attrition rate >20%, single-country study, only presented aggregate bleeding data for 0–6 and 0–12 month intervals	Fair/high
Gao et al (1990)^d^^,^^e^	Randomized comparative trial; China; 1989–90; compared clinical performance, contraceptive efficacy and acceptability of the LNG-IUS and Norplant-2 implants (Schering Oy, Berlin, Germany) during the first year of use	100 LNG-IUS and 100 Norplant-2 users; healthy parous women, aged 20–40 years, requesting contraception; not breast-feeding, normal pelvic examination, blood hemoglobin level at least 11 g/dL; no injectable steroid hormones or oral contraceptives during preceding 6 months	3.1 (3/97)^d^		—	—	18.9 (17/90)	Prospective trial, few exclusion criteria, used validated tool for diaries, attrition <20	Single-country study; only presented aggregate bleeding data for 0–6 and 0–12 month intervals	Good/low
Gemzell-Danielsson et al (2012)	Randomized, open-label, 3-arm, phase II study conducted in Finland, Sweden, Norway, Hungary, United Kingdom; 2005–2008; aimed to identify an appropriate daily dose for a new, smaller, lower dose LNG-IUS suitable for nulliparous and parous women	738 women; 284 lower-dose users and 254 LNG-IUS 20 (Mirena) users; ages 21–40 years; followed for 3-years afterinsertion; sexually-active women requesting contraception and with regular menstrual cycles (21–35 days) were eligible; women were excluded if they were lactating, had given birth or terminated a pregnancy 12 weeks before screening, had a distorted uterine cavity, menorrhagia, previous ectopic pregnancy, PID, or other LNG-IUS contraindications; assessed completed bleeding diaries at 1, 6, and 12 months during first year, but solely presented data for second 90-day interval	—	5.9 (14/239)	—	—	—	Prospective data collection, multinational trial, used validated tool to collect bleeding data, randomized to comparative LARC groups, attrition rate <20%, long follow-up period	Only presented results for single interval. despite collecting over 12-month period	Good/low
Modesto et al (2014)	Randomized trial to evaluate impact of counseling on discontinuation; Campinas, Brazil; 2011–2013	99 LNG-IUS users and 198 starting other methods, ages 18–40 years; followed for 12 months afterinsertion; women attending the University of Campinas FP clinic and requesting any LARC method eligible to participate	0.0 (0/99)	7.4 (6/81)	14.7 (10/68)	38.2 (26/68)	—	Prospective trial, used validated tool to collect bleeding data, few exclusion criteria	Attrition rate >20%, single-country study, data graphically presented	Fair/high
Pakarinen et al (1997)	Randomized comparative trial conducted in Finland; 1996–1997; compared LNG-IUS to intracervical placement of levonorgestrel product	298 women; 147 intrauterine LNG-IUS users, 151 intracervical LNG-IUS users; ages 18–43 years; followed for 12 months afterinsertion; women with evidence of hormonal injectable/implant/or oral contraceptives use within 6 months of enrollment, current gynecologic infection or history of PID or salpingitis during the previous 12 months, previous ectopic pregnancy, pregnancy or breast-feeding, or any contraindications to LNG-IUS placement were excluded	0.9 (1/116)	7.3 (8/109)	10.9 (11/101)	16.0 (15/94)	—	Prospective trial, used externally validated tool to collect bleeding data, participants provided specific instructions on how to document bleeding/spotting events, few exclusion criteria, randomized participants to comparative groups, used allocation concealment	Attrition rate >20%, single-country study	Fair/high
Sivin et al (1987)	Randomized comparative trial conducted in United States, Brazil, Egypt, Chile, Singapore, Dominican Republic; 1982–1987; compared the overall performance of LNG-IUS and TCu 380Ag copper IUD	1124 LNG-IUS users and 1121 TCu 380Ag users; ages 18–38 years; followed for 5 years after insertion; women with a contraindication to copper IUDs or contraceptive steroids, with a history of PID after last pregnancy or a history of ectopic pregnancy were excluded	—	10.0 (74/743)	—	—	—	Prospective, multinational trial, used externally validated tool to collect bleeding data, large sample size, single-blinded, used allocation concealment to randomize participants to study groups, few exclusion criteria, long follow-up period	Assessed bleeding diaries at 1, 3, 6, and 12 months post LNG-IUS insertion, but only presented second-period interval, attrition rate >20%	Fair/high
Wang et al (1992)^e^	Randomized comparative trial; China; 1989–1990; compared clinical performance, contraceptive efficacy and acceptability of the LNG-IUS and Norplant-2 implants during the first year of use	100 LNG-IUS and 100 Norplant-2 users; healthy parous women, aged 20–40 years, requesting contraception; not breast-feeding, normal pelvic examination, blood hemoglobin level at least 11 g/dL; no injectable steroid hormones or oral contraceptives during preceding 6 months.	0.0 (0/100)	5.2 (5/97)	9.7 (9/93)	16.7 (15/90)	—	Prospective trial, few exclusion criteria, used validated tool for diaries, attrition <20%	Single country study, data graphically presented	Fair/low

*FP*, family planning; *IUD*, intrauterine device; *LARC*, long-acting reversible contraception; *LNG-IUS*, levonorgestrel intrauterine system; *PID*, pelvic inflammatory disease.

*Sergison and Maldonado. Amenorrhea associated with LNG-IUS use. Am J Obstet Gynecol 2019*.

a Bias (low, medium, high) assessment was based on likelihood of attrition bias (if attrition <10%=low, 10–20%=medium, >20% high risk of bias); quality (good, fair, poor) assessment was based on the following criteria: study location (multiple vs single country), data presentation (tabular vs graphic depiction of point estimate), and the consistency of interval data provided (i.e. all four or only some 90-day interval data provided); sample size was controlled for in the metaanalysis and thus not considered in critical appraisal process

b USPSTF. *Procedure manual*. 2015

c Cochrane. Assessing Risk of Bias in Included Studies. Cochrane Methods Bias 2018. Available at: https://methods.cochrane.org/bias/assessing-risk-bias-included-studies. Accessed January 31, 2018

d 0–6 month estimates not included in metaanalysis because of limited number of articles

e Distinct analyses of the same study cohort.

## Appendix A

### Appendix A1 Full PubMed search strategy

(Intrauterine Devices, Medicated[Mesh] OR “intrauterine system” OR Mirena[tiab] OR Levosert[tiab] OR Liletta[tiab] OR “levonorgestrel” OR “LNG” OR “levonorgestrel-releasing” OR “levonorgestrel releasing” AND “IUD*” OR “IUCD” OR “IUS” OR “intrauterine device” OR “intrauterine devices” OR “intrauterine system” OR “intrauterine systems” OR “intrauterine contraceptive device” OR “intrauterine contraceptive devices” AND “bleeding” OR “spotting” OR “menstrual” OR “menstruation” OR “amenorrh*” OR “amenorrhea” OR “bleeding pattern” OR (“menstruation” AND “suppression”) OR (“bleeding” AND suppression”) OR “menstrual disturbance” OR “menstrual changes”) AND (Clinical Trial[ptyp] OR Comparative Study[ptyp] OR Clinical Study[ptyp] OR Evaluation Studies[ptyp])

### Appendix A2 Formula for the calculation of precision-based (inverse variance) weights

$$w_{i}^{*}=\frac{1}{v_{i}+\tau^{2}}$$

Where $w_{i}^{*}$ is the calculated weightl $v_{i}$ is the intrastudy variance per study (i), and $\tau^{2}$ is the interstudy variance per 90-day interval.

## References

[1] Luukkainen T., Allonen H., Haukkamaa M., Lahteenmaki P., Nilsson C.G., Toivonen J. (1986). Five years’ experience with levonorgestrel-releasing IUDs. Contraception.

[2] Belsey E.M., Machin D., D’Arcangues C. (1986). The analysis of vaginal bleeding patterns induced by fertility regulating methods: World Health Organization Special Programme of Research, Development and Research Training in Human Reproduction. Contraception.

[3] Den Tonkelaar I., Oddens B.J. (1999). Preferred frequency and characteristics of menstrual bleeding in relation to reproductive status, oral contraceptive use, and hormone replacement therapy use. Contraception.

[4] Peipert J.F., Zhao Q., Allsworth J.E. (2011). Continuation and satisfaction of reversible contraception. Obstet Gynecol.

[5] Lin K., Barnhart K. (2007). The clinical rationale for menses-free contraception. J Womens Health (Larchmt).

[6] Chrisman C., Ribeiro P., Dalton V.K. (2007). The levonorgestrel-releasing intrauterine system: an updated review of the contraceptive and noncontraceptive uses. Clin Obstet Gynecol.

[7] (2017). LILETTA (levonorgestrel-releasing intrauterine system) 52 mg [prescribing information].

[8] (2017). MIRENA (levonorgestrel-releasing intrauterine system) 52 mg [prescribing information].

[9] Moher D., Liberati A., Tetzlaff J., Altman D.G., PRISMA Group (2009). Preferred reporting items for systematic reviews and meta-analyses: the PRISMA statement. BMJ.

[10] Higgins J.P., Green S. (2011). Cochrane handbook for systematic reviews of interventions, version 5.1.0.

[11] Perez A., Vela P., Masnick G.S., Potter R.G. (1972). First ovulation after childbirth: the effect of breast-feeding. Am J Obstet Gynecol.

[12] Perez A., Vela P., Potter R., Masnick G.S. (1971). Timing and sequence of resuming ovulation and menstruation after childbirth. Popul Stud (Camb).

[13] Jarvela I., Tekay A., Jouppila P. (1998). The effect of a levonorgestrel-releasing intrauterine system on uterine artery blood flow, hormone concentrations and ovarian cyst formation in fertile women. Hum Reprod.

[14] Andersson K., Batar I., Rybo G. (1992). Return to fertility after removal of a levonorgestrel-releasing intrauterine device and Nova-T. Contraception.

[15] Mejia M., McNicholas C., Madden T., Peipert J.F. (2016). Association of baseline bleeding pattern on amenorrhea with levonorgestrel intrauterine system use. Contraception.

[16] Lethaby A., Hussain M., Rishworth J.R., Rees M.C. (2015). Progesterone or progestogen-releasing intrauterine systems for heavy menstrual bleeding. Cochrane Database Syst Rev.

[17] Qiu J., Cheng J., Wang Q., Hua J. (2014). Levonorgestrel-releasing intrauterine system versus medical therapy for menorrhagia: a systematic review and meta-analysis. Med Sci Monit.

[18] Bitzer J., Heikinheimo O., Nelson A.L., Calaf-Alsina J., Fraser I.S. (2015). Medical management of heavy menstrual bleeding: a comprehensive review of the literature. Obstet Gynecol Surv.

[19] US Preventive Services Task Force (2015). Procedure manual.

[20] Cochrane (2018). Assessing Risk of Bias in Included Studies. Cochrane Methods Bias.

[21] Borenstein M., Hedges L.V., Higgins J.P., Rothstein H.R. (2010). A basic introduction to fixed-effect and random-effects models for meta-analysis. Res Synth Methods.

[22] Simmonds M.C., Higgins J.P., Stewart L.A., Tierney J.F., Clarke M.J., Thompson S.G. (2005). Meta-analysis of individual patient data from randomized trials: a review of methods used in practice. Clin Trials.

[23] Nilsson C.G., Luukkainen T., Diaz J., Allonen H. (1982). Clinical performance of a new levonorgestrel-releasing intrauterine device: a randomized comparison with a nova-T-copper device. Contraception.

[24] Dubuisson J.B., Mugnier E. (2002). Acceptability of the levonorgestrel-releasing intrauterine system after discontinuation of previous contraception: results of a French clinical study in women aged 35 to 45 years. Contraception.

[25] Gao J., Wang S.L., Wu S.C., Sun B.L., Allonen H., Luukkainen T. (1990). Comparison of the clinical performance, contraceptive efficacy and acceptability of levonorgestrel-releasing IUD and Norplant-2 implants in China. Contraception.

[26] Wang S.L., Wu S.C., Xin X.M., Chen J.H., Gao J. (1992). Three years’ experience with levonorgestrel-releasing intrauterine device and Norplant-2 implants: a randomized comparative study. Adv Contracept.

[27] Darney P.D., Stuart G.S., Thomas M.A., Cwiak C., Olariu A., Creinin M.D. (2018). Amenorrhea rates and predictors during 1 year of levonorgestrel 52 mg intrauterine system use. Contraception.

[28] Modesto W., Bahamondes M.V., Bahamondes L. (2014). A randomized clinical trial of the effect of intensive versus non-intensive counselling on discontinuation rates due to bleeding disturbances of three long-acting reversible contraceptives. Hum Reprod.

[29] Pakarinen P.I., Suvisaari J., Luukkainen T., Lahteenmaki P. (1997). Intracervical and fundal administration of levonorgestrel for contraception: endometrial thickness, patterns of bleeding, and persisting ovarian follicles. Fertil Steril.

[30] Gemzell-Danielsson K., Schellschmidt I., Apter D. (2012). A randomized, phase II study describing the efficacy, bleeding profile, and safety of two low-dose levonorgestrel-releasing intrauterine contraceptive systems and Mirena. Fertil Steril.

[31] Sivin I., Stern J., Diaz J. (1987). Two years of intrauterine contraception with levonorgestrel and with copper: a randomized comparison of the TCu 380Ag and levonorgestrel 20 mcg/day devices. Contraception.

[32] Andersson K., Odlind V., Rybo G. (1994). Levonorgestrel-releasing and copper-releasing (Nova T) IUDs during five years of use: a randomized comparative trial. Contraception.

[33] Koh S.C., Singh K. (2010). Levonorgestrel-intrauterine system effects on hemostasis and menstrual blood loss in women seeking contraception. J Obstet Gynaecol Res.

[34] Weisberg E., Bateson D., McGeechan K., Mohapatra L. (2014). A three-year comparative study of continuation rates, bleeding patterns and satisfaction in Australian women using a subdermal contraceptive implant or progestogen releasing-intrauterine system. Eur J Contracept Reprod Health Care.

[35] Stone A.A., Shiffman S., Schwartz J.E., Broderick J.E., Hufford M.R. (2003). Patient compliance with paper and electronic diaries. Control Clin Trials.

[36] Nippita S., Oviedo J.D., Velasco M.G., Westhoff C.L., Davis A.R., Castano P.M. (2015). A randomized controlled trial of daily text messages versus monthly paper diaries to collect bleeding data after intrauterine device insertion. Contraception.

[37] World Health Organization (1983). Patterns and perceptions of menstruation.

[38] Hubacher D., Chen P.L., Park S. (2009). Side effects from the copper IUD: do they decrease over time?. Contraception.

[39] Machin D., Farley T.M., Busca B., Campbell M.J., D’Arcangues C. (1988). Assessing changes in vaginal bleeding patterns in contracepting women. Contraception.

[40] Eisenberg D.L., Schreiber C.A., Turok D.K. (2015). Three-year efficacy and safety of a new 52-mg levonorgestrel-releasing intrauterine system. Contraception.

[41] Schreiber C.A., Teal S.B., Blumenthal P.D., Keder L.M., Olariu A.I., Creinin M.D. (2018). Bleeding patterns for the Liletta® levonorgestrel 52 mg intrauterine system. Eur J Contracept Reprod Health Care.

[42] Mulrow C.D. (1994). Rationale for systematic reviews. BMJ.

[43] Cornish F. (2015). Evidence synthesis in international development: a critique of systematic reviews and a pragmatist alternative. Anthropol Med.

[44] Schmidt E.O., James A., Curran K.M., Peipert J.F., Madden T. (2015). Adolescent experiences with intrauterine devices: a qualitative study. J Adolesc Health.

[45] Brown M.K., Auerswald C., Eyre S.L., Deardorff J., Dehlendorf C. (2013). Identifying counseling needs of nulliparous adolescent intrauterine contraceptive users: a qualitative approach. J Adolesc Health.

[46] Backman T., Huhtala S., Tuominen J. (2001). Sixty thousand woman-years of experience on the levonorgestrel intrauterine system: an epidemiological survey in Finland. Eur J Contracept Reprod Health Care.

